# Serum Electrolytes in Patients Presenting With Acute Exacerbation of Chronic Obstructive Pulmonary Disease (COPD) and Their Comparison With Stable COPD Patients

**DOI:** 10.7759/cureus.38080

**Published:** 2023-04-24

**Authors:** Akash Deep, Prashanta R Behera, Saswat Subhankar, Aarthi Rajendran, C Mohan Rao

**Affiliations:** 1 Respiratory Medicine, Kalinga Institute of Medical Sciences, Bhubaneswar, IND; 2 Critical Care, Kalinga Institute of Medical Sciences, Bhubaneswar, IND

**Keywords:** acute exacerbations, chronic obstructive pulmonary disease, hyponatremia, mortality, morbidity, electrolytes, copd

## Abstract

Introduction

Chronic obstructive pulmonary disease (COPD) is a major cause of morbidity and mortality, which may be further aggravated by episodes of acute exacerbation of COPD (AECOPD). Electrolyte imbalances during these episodes may add to the duration of hospitalization and disease outcome.

Aims and objectives

This study aims to compare the serum electrolyte levels of patients with AECOPD and stable COPD and correlate them with the severity of exacerbation and disease outcome.

Materials and methods

The study was conducted as a case-control study between January 2021 and December 2022. Patients with AECOPD and stable COPD were included as “cases” and “controls,” respectively. The various serum electrolyte levels were defined as per recent guidelines. Statistical analysis was performed using SPSS 20.0 (IBM Corp., Armonk, NY).

Results

A total of 75 patients were included with 41 in the study group and 34 in the control group. The majority of people were between the ages of 61 and 70. The most frequent electrolyte abnormality found was hyponatremia. The mean serum sodium and calcium levels were lower in patients with AECOPD while the mean serum potassium levels were higher. A total of five deaths were recorded in patients with two or more electrolyte imbalances. The latter also had a requirement for home oxygen or non-invasive ventilation at the time of discharge.

Conclusion

Patients of AECOPD with multiple electrolyte imbalances need scrutinized treatment as they are more prone to develop complications, have poorer outcomes, and prolonged hospital stays.

## Introduction

Chronic obstructive pulmonary disease (COPD) in general terms pertains to the chronic outflow obstruction that develops due to various reasons, most commonly due to chronic tobacco smoking. The Global Initiative for Chronic Obstructive Lung Disease (GOLD) 2023 has defined COPD as follows:

“COPD is a heterogeneous lung condition characterized by chronic respiratory symptoms (dyspnea, cough, sputum production, exacerbations) due to abnormalities of the airways (bronchitis, bronchiolitis) and/or alveoli (emphysema) that cause persistent, often progressive airflow obstruction” [1].

Acute exacerbations of COPD (AECOPD) are important episodes in the natural history of the disease as they adversely affect health status, rate of hospitalization, and disease progression. Various metabolic disorders have been associated with patients of COPD, especially AECOPD. These disorders include hyponatremia, hypokalemia, hypocalcemia, hypomagnesemia, hypophosphatemia, elevated liver enzymes, and uric acid levels, and amongst them, hyponatremia and hypokalemia are the most commonly reported.

While hyponatremia develops due to causes like chronic hypoxia, hypercapnia, heart failure, or syndrome of inappropriate antidiuretic hormone secretion (SIADH), hypokalemia primarily develops due to metabolic alkalosis or long-term beta-2 agonist and steroid use [2,3]. Although these are mostly correctible, they are usually overlooked, thus confusing the diagnosis and adding to overall morbidity and mortality.

Aims and objectives

Primary Objective

To determine the serum electrolyte levels (sodium, potassium, magnesium, chloride, and calcium) in patients admitted as AECOPD and to compare the values of serum electrolytes between patients of AECOPD and stable COPD.

Secondary Objective

To correlate the levels of serum electrolytes with disease outcomes.

## Materials and methods

It was a case-control study conducted in the Department of Respiratory Medicine, Kalinga Institute of Medical Sciences, Bhubaneswar, during the period from January 2021 to December 2022. All consecutive patients with a prior diagnosis of COPD who visited the pulmonary or general medicine OPD or emergency department were taken as cases and controls. Patients who had pneumonia, tuberculosis, neoplastic etiologies, and hepatic, cardiac, and renal diseases were excluded.

Cases and controls

Cases

Patients with a diagnosis of AECOPD as defined by a case with a prior diagnosis of COPD who presents with an increase in respiratory symptoms (cough, expectoration, or breathlessness) that require a change in medications and/or hospital admission.

Controls

Patients with stable COPD defined as patients with a prior diagnosis of COPD who did not have any exacerbations in the last four weeks.

Sample size

By considering a two-sided confidence interval of 95%, power of 80%, the hypothetical proportion of controls with dyselectrolytemia as 20%, and the hypothetical proportion of cases with dyselectrolytemia as 58%, the number of cases and controls was determined to be 30 in each group (Fleiss method with continuity correction).

These patients were evaluated based on their clinical history and complete clinical examination, routine blood investigations, including serum electrolytes, and radiological evaluation with chest X-ray. All serum electrolyte levels were defined as per recent guidelines [4].

Statistical analysis

The presentation of continuous data was done using mean ± standard deviation (SD). The categorical values were represented as percentages and numerals. The Student's t-test was used to compare two distinct groups, and the chi-square test was used to compare proportions in different categories. Two proportions were compared using the z-test. When analyzing three or more variables, the Mann-Whitney U test was utilized.

All statistical analyses were performed using SPSS for Windows version 20.0 (IBM Corp., Armonk, NY). Statistical significance was defined as a p-value less than 0.05.

## Results

The mean age of the patients in the study group was 69.1 ± 10.36 years while that in the control group was 68.12 ± 9.95 years (p = 0.684). The male-to-female ratio in each group was 4.25:1 and 2.4:1, respectively. The majority of the patients belonged to the age group of 61-70 years (Table 1). Cough and breathlessness were the most commonly reported clinical features and hypertension was the most common comorbidity. The total leucocyte count was significantly raised in patients with AECOPD (Table 1). Based on the criteria by Burge and Wedzicha, the majority of patients in the study group had life-threatening exacerbation (36, 87.8%) (Table 2).

**Table 1:** Demography, clinical, and laboratory parameters P-value < 0.05 is significant.

Age distribution (years)	Cases (n = 41)	Control (n = 34)	p
<60	10	5	0.15
61-70	14	17	0.08
71-80	12	9	0.269
>80	5	3	0.3
Clinical features			
Cough	37	30	0.39
Breathlessness	32	28	0.32
Expectoration	19	17	0.37
Chest pain	4	0	0.03
Hemoptysis	2	0	0.095
Comorbidities			
Hypertension	7	5	0.39
Diabetes	1	1	0.45
Laboratory parameters			
Total leukocyte count	13.64 ± 6.23	10.04 ± 3.14	0.0003
Neutrophils	83.53 ± 7.64	79.29 ± 17.63	0.17
Serum urea	40.84 ± 23.57	33.48 ± 17.67	0.137
Serum creatinine	0.97 ± 0.31	0.89 ± 0.36	0.3
Serum bilirubin	0.75 ± 0.63	0.79 ± 0.4	0.697

**Table 2:** Severity of exacerbation

Severity	No. of patients
Mild	0
Moderate	0
Severe	3
Very severe	2
Life-threatening	36

Hyponatremia was the most commonly recorded dyselectrolytemia, which was seen in 29 (70.7%) patients with AECOPD and 16 (47.1%) patients with stable COPD. This was followed by hypocalcemia seen in 23 patients (56.1%) of the cases and nine patients (26.5%) of the controls (Table 3). Hyperkalemia was recorded only in patients with AECOPD (9, 22%) (Table 3).

**Table 3:** Comparison of serum electrolytes among study and control groups mmol/l: millimoles per liter; mg/dl: milligram per deciliter; cells/mm3: cells per cubic millimeter. P-value < 0.05 is significant.

Laboratory parameter			Group		p
			Study group	Control group	
Serum sodium (mmol/L)	Hypo	n	29	16	0.037
		%	70.7%	47.1%	
	Normal	n	12	18	
		%	29.3%	52.9%	
Serum potassium (mmol/L)	Hypo	n	12	11	0.013
		%	29.3%	32.4%	
	Normal	n	20	23	
		%	48.8%	67.6%	
	Hyper	n	9	0	
		%	22.0%	0.0%	
Serum calcium (mg/dl)	Hypo	n	23	9	0.006
		%	56.1%	26.5%	
	Normal	n	17	18	
		%	41.5%	52.9%	
	Hyper	n	1	7	
		%	2.4%	20.6%	
Serum phosphate (mg/dl)	Hypo	n	5	3	0.893
		%	12.2%	8.8%	
	Normal	n	30	26	
		%	73.2%	76.5%	
	Hyper	n	6	5	
		%	14.6%	14.7%	
Serum magnesium (mg/dl)	Hypo	n	7	8	0.764
		%	17.1%	23.5%	
	Normal	n	34	26	
		%	82.9%	76.5%	

The mean serum sodium level was lower in patients with AECOPD (129.39 ± 8.654 meq/L) as compared to stable COPD patients (133.71 ± 5.374 meq/L). Similarly, the serum calcium levels were also lower in the study group. The serum potassium level was higher in patients with AECOPD (4.1463 ± 3.94) than in stable COPD patients (3.49 ± 0.619). The levels of phosphate and magnesium were comparable in both groups (Table 4).

**Table 4:** Comparison of mean serum electrolyte levels among study and control groups mg/dl: milligram per deciliter; cells/mm3: cells per cubic millimeter. P-value < 0.05 is significant.

Laboratory parameter		Mean	Std. deviation	p
Serum sodium (mmol/L)	Study group	129.39	8.654	0.014
	Control group	133.71	5.374	
Serum potassium (mmol/L)	Study group	4.1463	1.08837	0.333
	Control group	3.9412	0.61993	
Serum calcium (mg/dl)	Study group	8.437	0.8904	0.001
	Control group	9.297	1.3111	
Serum phosphate (mg/dl)	Study group	3.554	1.0726	0.960
	Control group	3.565	0.7702	
Serum magnesium (mg/dl)	Study group	2.029	0.3919	0.820
	Control group	2.009	0.3769	

The mean duration of hospitalization in the study group was 11.56 ± 10.79 days. A total of three (7.3%) patients required only oxygen inhalation, 25 (60.96%) required non-invasive ventilation (NIV), and six (14.6%) required invasive ventilation. Seven (17.07%) deaths were recorded in the study group. A total of 34 (82.93%) patients were discharged, and 10 (24.4%) patients were discharged with home NIV and oxygen support.

The serum electrolytes were lower in patients who died when compared to those who survived. The values were found to be statistically significant for sodium and potassium. A total of five (14.6%) deaths were recorded in patients who had two or more electrolyte imbalances (p = 0.0548). The mean levels of serum sodium, potassium, and calcium were significantly lower in patients who died than those who survived (p = 0.0001 and 0.009, respectively) (Table 5); nine (21.95%) patients with two or more electrolyte disturbances required home oxygen therapy or NIV support (p = 0.003).

**Table 5:** Comparison of serum electrolytes and outcome in the study group mmol/l: millimoles per liter; mg/dl: milligrams per deciliter.

Electrolyte	Survivors (n = 34)	Deaths (n = 7)	p
Serum sodium (mmol/L)	131.59 ± 6.53	118.71 ± 10.23	0.0001
Serum potassium (mmol/L)	4.34 ± 0.96	3.189 ± 1.24	0.009
Serum calcium (mg/dl)	8.485 ± 0.86	8.2 ± 1.047	0.44

## Discussion

According to many systematic reviews and meta-analyses, COPD is more common in smokers or ex-smokers, men, and those who are above 40 years of age [5,6]. The PLATINO study determined the prevalence of post-bronchodilator airflow obstruction in Brazil, Uruguay Chile, Mexico, and Venezuela. It reported the prevalence to be highest in those >60 years of age [7].

Cough and breathlessness were the most common symptoms reported in our study in both groups. Progressive dyspnea and cough are the usual symptoms at presentation in COPD patients and an increase in these symptoms is seen during an acute exacerbation. Cough in COPD has been attributed to various factors like airway inflammation and excessive mucus production, production of mediators that are tussive (e.g. prostaglandins), and decreased threshold of cough receptors [8]. Similarly, shortness of breath is also multifactorial; dynamic hyperinflation, increased ventilatory demand, hypoxemia, hypercapnia, and neuromechanical dissociation are the possible factors. This has been reported in previous studies as well [9].

Hemoptysis is not very uncommon in patients suffering from AECOPD and many times investigation may fail to reveal an accurate etiology. However, it has been reported as a symptom in patients with COPD [10]. In our study, 4.88% of patients had complaints of hemoptysis.

Hypertension and diabetes mellitus were the commonest comorbidities reported in our study. Cardiovascular comorbidities are common in patients suffering from COPD. This is attributed to various factors like endothelial dysfunction and coagulopathy [11]. An important role is being played by systemic inflammation in the development of insulin resistance. This risk is higher in smokers [12].

In our study, 19 (46%) patients with AECOPD had a raised total leucocyte count (TLC), suggesting an infective etiology. The mean TLC count was considerably higher in patients with AECOPD (p = 0.003). The mean neutrophil count was also higher in patients with AECOPD, although not statistically significant (p = 0.17). Neutrophil counts have been previously associated with a greater number of severe exacerbations. The patients in the study group also had higher serum urea and creatinine levels. During exacerbation of COPD, the impaired gaseous exchange and carbon dioxide retention can reduce the renal blood flow and thus decrease the GFR. This is more prevalent in the presence of comorbidities like hypertension, diabetes mellitus, and atrial fibrillation [13].

The mean serum bilirubin levels were lower in the AECOPD patients (p = 0.097). A lower bilirubin level is associated with a higher risk of exacerbations [14]. The median levels of enzymes aspartate aminotransferase (AST) and alanine aminotransferase (ALT) were also lower in the study group (p = 0.019 and 0.037, respectively). Low ALT levels have, however, been associated with sarcopenia and thus poor long-term survival in AECOPD patients. However, other studies reported higher liver enzymes in patients with AECOPD at the time of presentation and this has been attributed to hypoxic hepatic damage [15].

Electrolyte imbalances are common in patients with AECOPD and add up to the overall morbidity and mortality. Hyponatremia was the main electrolyte imbalance in our study followed by hypokalemia. This was in accordance with previous studies [16,17]. COPD patients are prone to hyponatremia due to various reasons like the presence of worsening hypoxia, respiratory acidosis due to hypercapnia, right heart failure, SIADH, use of diuretics, and poor nutrition [2]. Hypokalemia in COPD may occur alone or in conjugation with hyponatremia. This develops due to factors like respiratory acidosis or metabolic alkalosis, chronic steroid use, and the use of β2 agonists like salbutamol [3].

Magnesium is an important element in bronchodilation and contraction of respiratory smooth muscle. Low magnesium during COPD exacerbation has been related to steroid use, use of beta-agonists, diuretics, and low dietary intake. Previous studies have also published similar results [18]. Hypocalcemia in COPD has mostly been correlated to vitamin D deficiency in these patients. Low serum calcium has been reported by many researchers [16]. Of the cases in our study, 56.1% had hypocalcemia as well as hypomagnesemia, although the differences observed between the groups were insignificant.

Phosphorous is an important element in all cells for the production of adenosine triphosphate (ATP). Low phosphorus in patients with COPD is associated with an increased need for ventilator support, duration of hospitalization, and mortality [19]. Our study reported a slightly lower level of phosphate in the study group (p = 0.960).

Among the patients in our study group who survived, 10 (24.4%) required home oxygen or NIV support; nine (90%) patients had two or more electrolyte imbalances. The mean serum electrolyte levels were lower in patients who required O2/NIV, although it was not statistically significant. In the study group, seven (17.07%) patients died; six patients had ≥2 electrolyte imbalances. All seven (100%) patients had hyponatremia, five (71.4%) had hypokalemia, four (57.1%) had hypocalcemia, and one (14.3%) had hypomagnesemia. Previous studies have found lower levels of electrolytes (sodium, potassium, magnesium, phosphorous, and calcium) in patients who died as compared to those who survived, although the correlation was statistically significant only in the case of potassium. This has also been supported by other researchers. However, a study by Lindner et al. did not find any association between disorders of sodium and potassium and the need for ICU admission or mortality [17,20].

Limitation of study

The study was a single-center study and had a small sample size. A similar study from other institutions is desirable. All consecutive cases were taken, so randomization and matching were also not done.

## Conclusions

Hyponatremia was the commonest electrolyte imbalance in patients with AECOPD followed by hypocalcemia and hypokalemia. Patients with two or more electrolyte imbalances required long-term oxygen therapy or NIV at the time of discharge and also had higher mortality. Hyponatremia and hypokalemia are of special importance since they are associated with many harmful effects, including dysrhythmia, central nervous system dysfunction, impaired nerve-muscle conduction, respiratory muscle dysfunction, and more. These and other electrolyte imbalances thus need to be monitored and promptly corrected to prevent fatal outcomes.
